# OpenChrom: a cross-platform open source software for the mass spectrometric analysis of chromatographic data

**DOI:** 10.1186/1471-2105-11-405

**Published:** 2010-07-30

**Authors:** Philip Wenig, Juergen Odermatt

**Affiliations:** 1 Department of Wood Science, University of Hamburg, Leuschnerstraße 91, 21031 Hamburg, Germany

## Abstract

**Background:**

Today, data evaluation has become a bottleneck in chromatographic science. Analytical instruments equipped with automated samplers yield large amounts of measurement data, which needs to be verified and analyzed. Since nearly every GC/MS instrument vendor offers its own data format and software tools, the consequences are problems with data exchange and a lack of comparability between the analytical results. To challenge this situation a number of either commercial or non-profit software applications have been developed. These applications provide functionalities to import and analyze several data formats but have shortcomings in terms of the transparency of the implemented analytical algorithms and/or are restricted to a specific computer platform.

**Results:**

This work describes a native approach to handle chromatographic data files. The approach can be extended in its functionality such as facilities to detect baselines, to detect, integrate and identify peaks and to compare mass spectra, as well as the ability to internationalize the application. Additionally, filters can be applied on the chromatographic data to enhance its quality, for example to remove background and noise. Extended operations like do, undo and redo are supported.

**Conclusions:**

OpenChrom is a software application to edit and analyze mass spectrometric chromatographic data. It is extensible in many different ways, depending on the demands of the users or the analytical procedures and algorithms. It offers a customizable graphical user interface. The software is independent of the operating system, due to the fact that the Rich Client Platform is written in Java. OpenChrom is released under the Eclipse Public License 1.0 (EPL). There are no license constraints regarding extensions. They can be published using open source as well as proprietary licenses. OpenChrom is available free of charge at http://www.openchrom.net.

## Background

Software has become an integral part of analysis techniques. Especially in the area of gas chromatography/mass spectrometry, automatic samplers enable high throughput analyses. Software assists handling large amounts of data generated by automated and fast operating analytical instruments. Modern computer systems are inexpensive, powerful and allow analysis techniques that could not have been applied in the past. Deconvolution, a chromatographic quality enhancing technique, demonstrates for instance that increasing processor power makes new analysis techniques applicable. The technique of deconvolution has been described by Biller and Biemann [1,2], Dromey et al. [3], Colby [4], Hindmarch et al. [5], Halket et al. [6], Kong et al. [7], Taylor et al. [8], Pool et al. [9,10] and Davies [11] in various ways. Stein [12] published an enhanced deconvolution algorithm that has been implemented in the software AMDIS (Automated Mass Spectral Deconvolution and Identification System) [13]. AMDIS is available free of charge from the National Institute of Standards and Technology (NIST). Windig et al. [14,15] described another approach to enhance chromatographic quality by a deconvolution method called CODA (Component Detection Algorithm). The commercially available software ACD/MS Manager [16] offers an implementation of this approach.

Increasing computational power enables new applications, but there is still a lack of interoperability. Instrument vendors, such as Agilent Technologies, Shimadzu, Thermo Fisher Scientific and Waters Corporation have created their own software and data format. Usually, the mass spectral data formats are binary and can only be accessed by the instrument vendors' proprietary software. Some commercial tools exist to convert the mass spectral data files into other formats, such as MASS Transit from PALISADE Corporation [17]. To avoid these limitations, some efforts have been made to design and implement interoperable data formats and software libraries as for example NetCDF [18] or mzXML [19,20]. But even if it is possible to convert the data files to other formats, there are drawbacks in data processing as each software implements specific functions, has its own graphical user interface and is in most cases commercially available only, as for example the applicable software of ChemStation, Xcalibur or MassLynx. Hence, the users are forced to become familiar with different software systems, user interfaces and methods. Moreover, the software tools primarily target only specific operating systems, such as Microsoft Windows and Mac OSX. The number of software applications that are independent of the operating system and can also be run under Unix or Linux is limited. Linux systems are open source, available at no cost and their usage increases in scientific research (see Scientific Linux [21]), as well as in the public sector [22,23]. Software applications, such as AMDIS, have been published to be used free of charge, but their source code is not disposable. Thus, it is not possible to evaluate the algorithms implemented in the software. Especially in the case of scientific research, it is not possible to figure them out and to extend them. Even if algorithms are described in published papers [2,4,9,12,24], it is often impossible to validate them manually due to the complexity of chromatographic data. Other applications like ChemStation, Xcalibur, ACD/MS Manager are proprietary and closed source. They are only commercially available. There is no means of revealing the correctness of their utilized algorithms. Efforts have been made to solve the problems of missing interoperability and restricted access to source codes and algorithms [25]. Bioclipse is a sophisticated project that is open source and is focused with its algorithms on metabolism analysis and gene sequencing. Its techniques are state-of-the-art. Some other projects are mMass [26], COMSPARI [27] and fityk [28], but they do have some restrictions regarding their interoperability and extensibility. BioSunMS [29] is a tool to read TOF (Time of Flight) mass spectral data files, but it is not able to read instrument vendors' native data files. The Chemistry Development Kit (CDK) [30] implements convenient features to edit chemical data and structures, but it has no appropriate user interface. The open source tool OpenMS [31] aims to edit mass spectrometric data, but it is not completely platform independent, as it is written in C++ programming language.

Projects like Bioclipse, Sashimi [32] or TPP (Trans-Proteomic Pipeline) [33] are focused on the evaluation of metabolism products and gene sequencing and make extensive use of accurate mass resolution techniques. But there is still a lack of software systems that are capable to enhance nominal mass spectral data files, that are flexible, extensible and that offer an easy to use graphical user interface. According to the authors' knowledge, no application offers functions to import vendor systems chromatographic data files and has the ability to edit and analyze chromatograms in the way ChemStation and AMDIS do. No application combines the flexibility in analyses, is easily extensible, open source, platform independent and has a configurable graphical user interface.

### Implementation

#### Architecture

**Figure 1:**
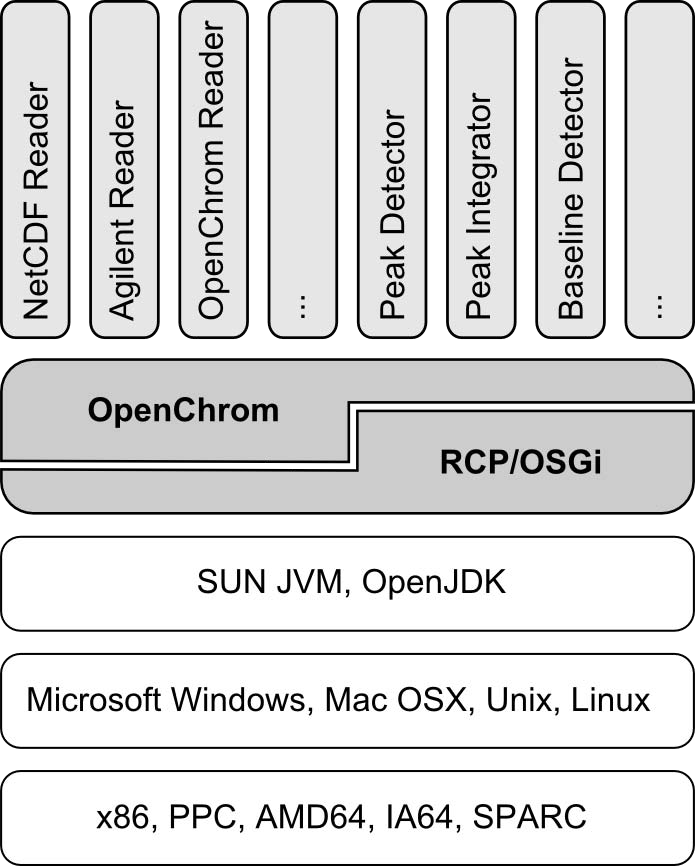
**RCP/OSGi and OpenChrom architecture**. The RCP/OSGi and OpenChrom architecture shows the supported processor platforms and operating systems.

OpenChrom is an open source software that aims to solve the aforementioned constraints getting rid of several restrictions. It is based on the Eclipse Rich Client Platform (RCP) [34], which is an OSGi (Open Service Gateway Initiative) based application environment that allows to build modular and flexible software systems. With the OSGi platform it is possible to extend the functionality of an application by dividing its components into different bundles. It is written in Java which is an interpreted language that depends on the Java Virtual Machine (JVM) and allows the execution of the software on several operating systems (Microsoft Windows, Mac OSX, Unix, Linux) and processor platforms (x86, PPC, AMD64, IA64, SPARC). It utilizes SWT (Standard Widget Toolkit) to render its graphical user interface by using the native resources of the underlying operating system. The Rich Client Platform is state-of-the-art in today's software development. The platform is open to be extended afterwards due to the chosen concepts. It means that the platform doesn't need to be full-fledged at the beginning. Further methods and implementations can be developed separately. Nonetheless, still some effort is necessary to design a platform that covers all needs of a software application to edit, evaluate and modify chromatographic data. In contrast to Bioclipse, Sashimi or TPP, OpenChrom has a slightly different scope, as it is focused primarily on nominal mass resolution data. Mass spectrometers for nominal mass resolution are inexpensive, as for example quadrupole or ion trap instruments. But the data acquisition limits the range of possible applications. Software has the potential to enhance the quality of the recorded data, in contrary to the given limitations. Hence, the Rich Client Platform and the Java programming language were chosen, as they offer an excellent support for a highly extensible and abstract base framework. The OSGi based Rich Client Platform Equinox supports the definition of extension points. The use of different class paths makes it possible to execute code from separated bundles (Figure 1). New functionality, e.g. to export a given chromatogram to a PDF file, can be implemented in a separate bundle making use of the extension point mechanism to import and export chromatographic data.

Tools in different areas have been implemented based on the Rich Client Platform, such as the Eclipse IDE (Integrated Development Environment), Lotus Notes, Bioclipse, BioSunMS, XMind, Apache Directory Studio and several more. It is part of the OpenChrom architecture to define useful extension points and to build a suitable object model.

### Object model

**Figure 2:**
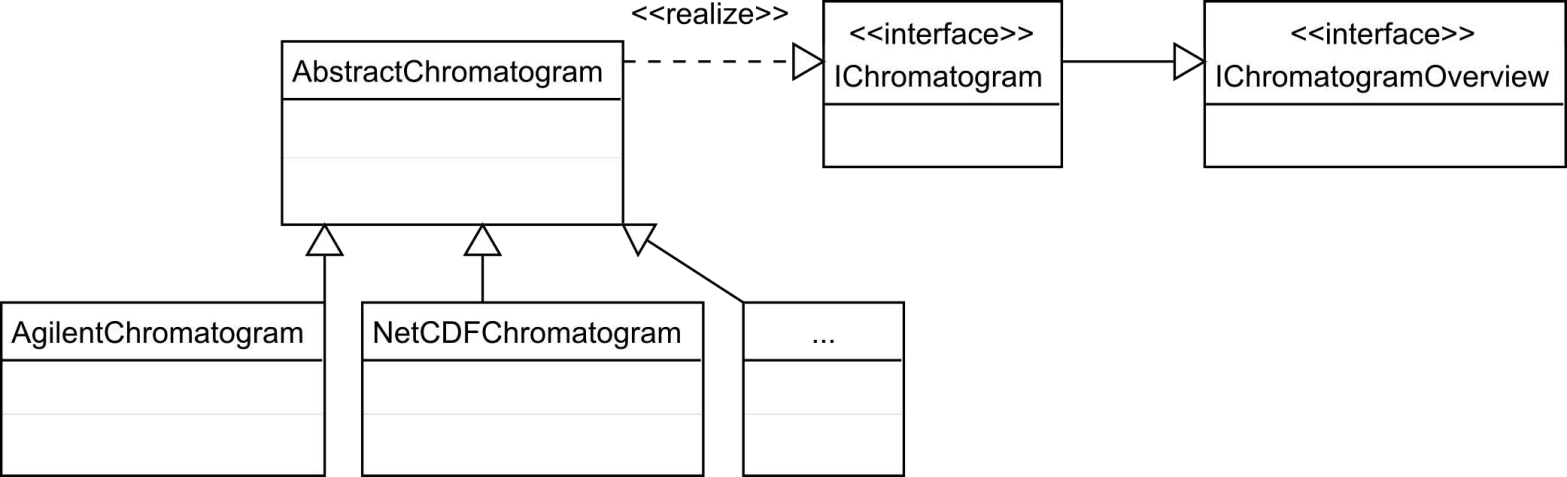
**OpenChrom chromatogram object model**. The OpenChrom chromatogram object model shows a simplified UML diagram of the chromatographic model OpenChrom uses.

OpenChrom provides a designed object model to define chromatograms, scans, mass spectra, peaks and baselines. It is important to abstract the base model, as it reduces dependencies in code and allows for the implemention of further extensions. Therefore, the decision was to support an enhanced chromatogram, mass spectrum and peak model, written in Java. There is no preliminary compilation necessary on different operating systems. Further on, it is possible to cover special needs regarding the import of instrument vendors' binary chromatographic files. An excerpt of the OpenChrom object model is shown in a simplified UML (Unified Modeling Language) diagram (Figure 2). Java, as an object orientated language, supports the use of the four base strategies in object orientation: abstraction, encapsulation, polymorphic behavior and inheritance [35]. OpenChrom makes extensive use of the object orientated concept. The interface "IChromatogram" and the abstract class "AbstractChromatogram" define and implement methods, which are common for all types of chromatograms, independent of the instrument vendors' data format. Therefore, it is not necessary to implement them iterative in each vendor specific chromatogram class. The base framework and extension points, like peak detectors and integrators, are working still with instances of the type "IChromatogram", instead of taking for example the differences of an Agilent and a NetCDF chromatogram into account. The object model for mass spectra and mass fragments, peaks and baselines is implemented in a similar way.

### Extension Points

The OpenChrom framework offers several bundles (Table 1). The most important one defines methods to implement specialized bundles that handle the import of chromatographic mass spectral data. It is possible to supply a bundle that is able to read binary chromatogram files, given by a specific instrument vendor. The bundle takes care of how to read a given file or directory. Furthermore, the framework offers extension points to detect and integrate peaks. The peak detection and integration have been separated, to make it possible to detect peaks with several peak detector methods and to integrate them with a specified integrator. This results in a more complex but also more flexible system. There is another extension point that allows to define bundles that are capable of detecting a baseline in the chromatogram model. Another flexible extension point was introduced, called filters. Bundles can extend the filter extension point to achieve a quality enhancement of the chromatographic data. They work comparable to filters in image processing software. One filter extension can for instance offer a set of methods to eliminate background signals from the chromatogram. Another filter can implement a routine to mean normalize the chromatogram. The filters offer editing steps, which are especially useful before peak detection and integration routines.

**Table 1:** Some selected bundles of the OpenChrom software.

Bundle	Description
baseline.detector	Detect baselines

comparison	Compare chromatograms and mass spectra

converter	Converter to read binary/textual data files

converter.supplier.agilent	Read Agilent data files

converter.supplier.cdf	Read and write NetCDF data files

...	...

filter	Modify chromatographic data

identifier	Identify chromatograms, mass spectra and peaks

integrator	Integrate peaks

model	Models (chromatogram, mass spectrum, peak,...)

peak.detector	Detect peaks

logging	Logging facility

rcp	Base application

thirdpartylibraries.*	Third party libraries (SWTChart, log4j,...)

...	...

The OpenChrom software offers several extension points. Extension points are declared in bundles. The table shows a selected overview of bundles and suppliers.

### Graphical User Interface

The Rich Client Platform offers a wide support to present an appropriate graphical user interface. Concepts detailing this include editors, views, perspectives, wizards, menus, cheat sheets, settings and help pages. OpenChrom makes extensive use of the available concepts. The editor shows the graphical representation of a chromatogram and several options, as for example a page to select or exclude distinct mass fragments. It also supports functions to save, edit and analyze chromatograms. The views are used to show different aspects of the chromatographic model. It is possible to show peaks in different kind of views. One view could show a peak including the background of the chromatogram. Another could show the peak with its increasing and decreasing tangents and its width at 50% height. A flexible mechanism was introduced to inform all views if the chromatogram selection has been changed. The update functionality is also realized by an extension point. Views and editors are composed in a task specific way using perspectives.

## Results and Discussion

**Figure 3:**
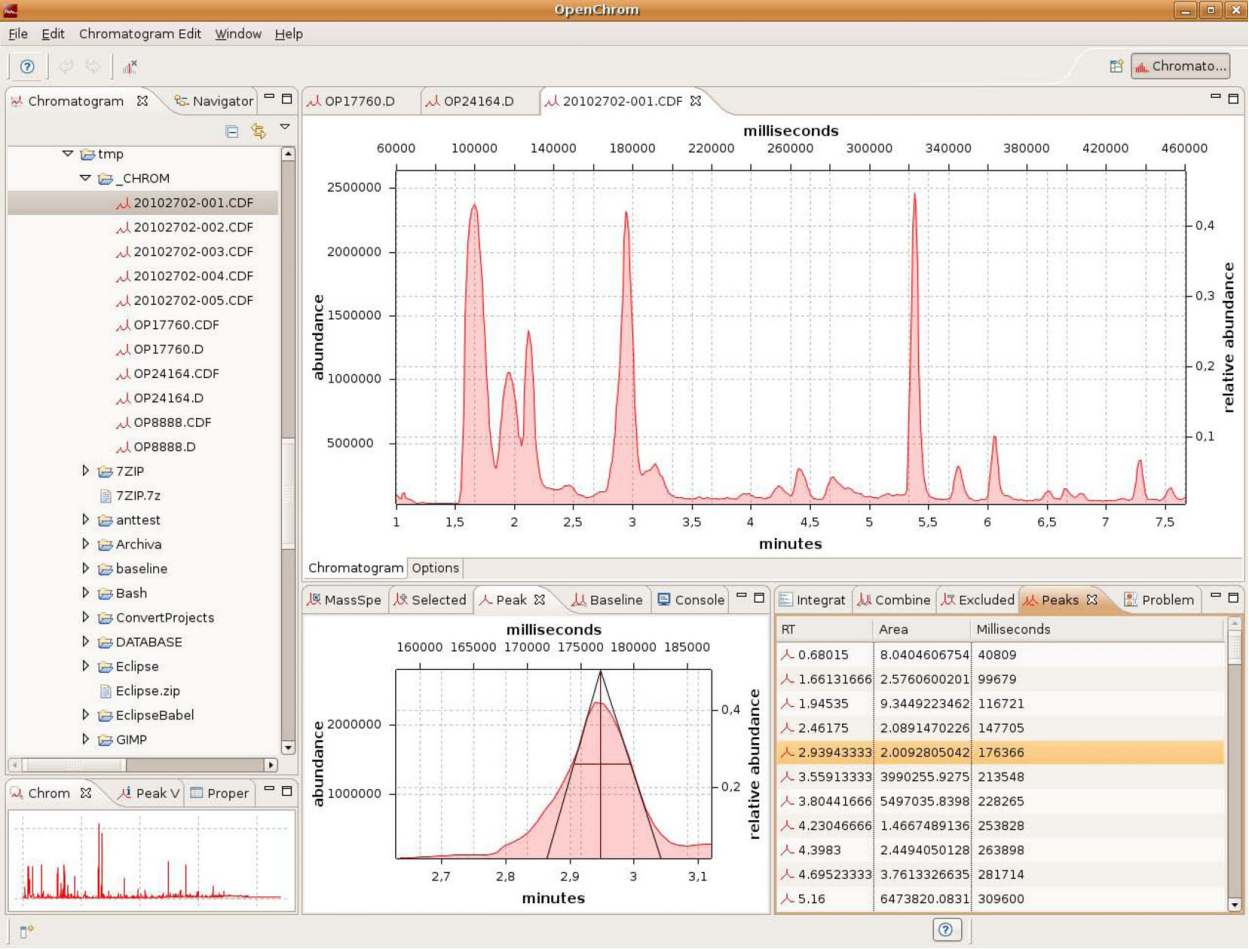
**OpenChrom software showing editors, views, menus and menu entries**. The OpenChrom software is using editors, views, menus and menu entries showed in the figure.

The OpenChrom software offers several options to edit and evaluate chromatographic data. It currently implements native converters to import mass spectrometric chromatograms from Agilent Technologies and to import and export NetCDF and mzXML files as well as a custom XML format to store the chromatographic data and additional information. The chromatogram file explorer (Figure 3) shows a representation of the local file system and marks those files and directories that contain importable chromatographic data files or directories. The chromatogram can either be stored in a file, a directory or a set of files, as the converter extension point and the import and export converters take care of it. The chromatogram will be opened by a double click on the file. Additionally, a preview of the selected chromatogram file is shown in a specialized view in the user interface. The chromatogram itself is shown in a multi-page editor that is divided into a chromatogram as well as an options page. It is possible to save the chromatogram in several file formats. The NetCDF, mzXML and the customized OpenChrom XML format are actually supported. Nonetheless, the time to import and to save a chromatogram depends on its format and size. It takes more time to process XML based formats like mzXML than binary formats like NetCDF or Agilents data format. The graphical elements are drawn using SWTChart and SWT. Chromatogram selections can be chosen by applying a "zoom in" or "zoom out" action in the chromatogram editor. All views will be updated after a zoom action.

The menu "Chromatogram Edit" allows to access functions that modify or evaluate the chromatographic data. For example, all registered bundles that support filters will be listed in the sub menu "Filter". It is possible to add a filter that implements a Savitzky-Golay [36] smoothing operation or to add filters that remove the background of the chromatogram. Each action will be performed on the active chromatogram selection. Actions are commonly very fast, due to the fact that the chromatogram is kept in the random access memory (RAM), depending on the implemented algorithms. Furthermore, the filter actions are reversible. This editing support is well known from modern IDEs and office suites. But the support for do/undo and redo operations does cost processing time. If the reversibility is not needed, it can be deactivated in the applications preference dialog. Another extension point is responsible to register baseline detectors. Different baseline detectors can be implemented in separated bundles and will be offered in the "Baseline Detectors" sub menu. Peak detection and integration are done commonly in one run. One improvement achieved through OpenChrom is a division of the detection and the integration of peaks into two separated actions. The peak detectors can be applied by calling an appropriate detector in the sub menu "Peak Detectors" and the peak integration can be performed by using an listed integrator from the sub menu "Integrators". The separation of detector and integrator methods makes it possible to detect peaks in a chromatogram using several algorithms and methods. The chosen peak detectors could be of different types, as for example detectors using deconvolution techniques like AMDIS or CODA. All detected peaks can afterwards be integrated by a unique integrator, which leads to comparable results. This feature offers a high flexibility in using different kinds of detectors and integrators.

**Figure 4:**
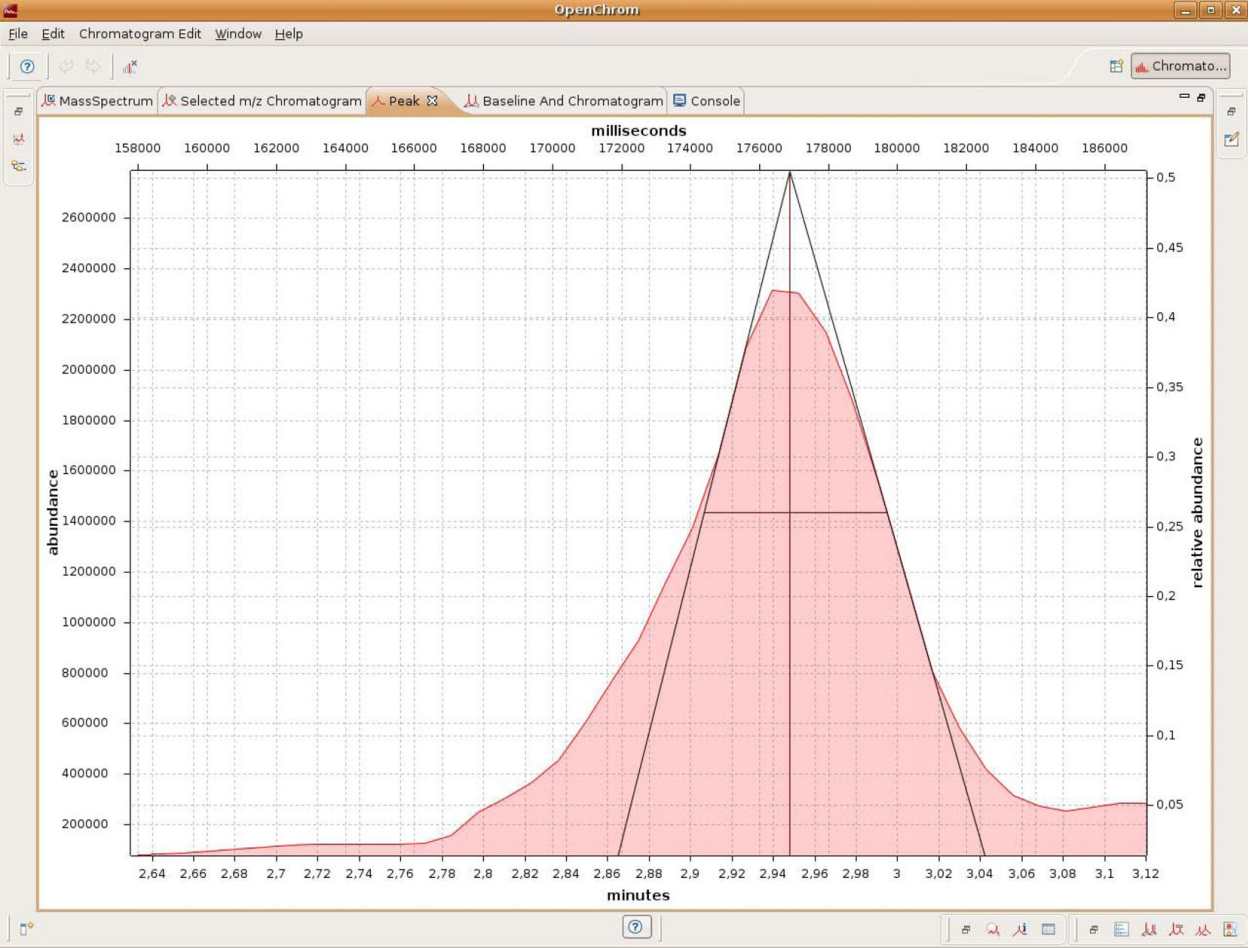
**Peak with increasing and decreasing tangents and its width at 50% height in extended format**. The view shows a maximized version of a selected peak.

**Figure 5:**
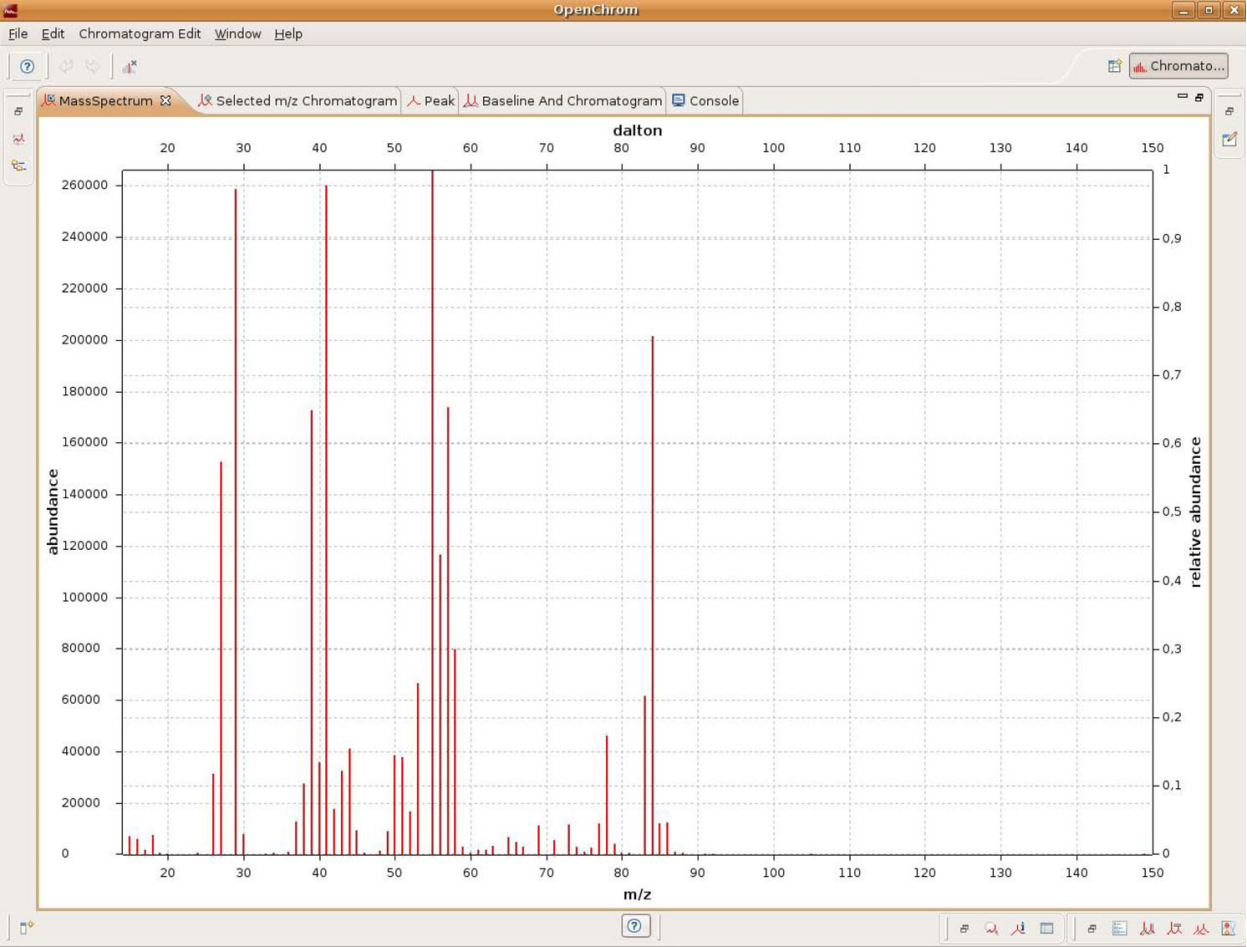
**Graphical representation of a mass spectrum in extended format**. The view shows a maximized version of a selected mass spectrum.

The view mechanism of the Eclipse Rich Client Platform makes it possible to show chromatographic data in different kind of views. A peak can be displayed in multiple ways, for example by its area (Figure 4), its increasing and decreasing tangents and its width at 50% of peak height. Thus, the system provides additional graphical information, especially useful for educational purposes. Each view can be shown in a small (Figure 3) and extended format (Figure 4 and 5), which allows an appropriate user interaction even on small displays.

Further on, property views show miscellaneous values of the selected chromatogram. Due to the chromatogram object model, different values will be shown if different chromatogram files have been loaded. Chromatograms from Agilent Technologies and NetCDF differ in their information content. Hence, the properties view helps to inspect the files. There are additional extension points implemented that enable adding bundles to compare mass spectra using different methods [24,37-40] or to identify peaks or chromatograms. A method similar to the one implemented in the software F-Search [41] from Frontier Laboratories Ltd. could be used to identify chromatograms, for example.

Moreover, the OpenChrom platform supports bundles with a system built-in logging mechanism that extends the Apache project log4j. Each module can use the logging mechanism which makes it easier to detect problems and failures. Bundles are further separated into fragments, which allows the separation of concerns. Each OpenChrom bundle supports an internationalization (i18n) and JUnit test fragment. At the moment, approximately 3000 unit tests are written and can be executed to ensure the quality of the software.

If necessary, the extension point mechanism gives the flexibility to add functions needed by users at any time. Thus, OpenChrom can be connected to other systems, as for example to LIMS (Laboratory Information Management System), databases, existing software tools or workflow systems. The object model of OpenChrom offers a convenient access to values and results from the edited chromatograms. Specialized modules take care of how to handle specific concerns, for example how to store results in an information management system. Further on, it is possible to implement bundles for specific analyses or for an automated experimentation.

OpenChrom enables several ways to edit and analyze chromatographic data. The advantage of the flexibility and the abstract architecture makes it partly difficult to get started with the platform, even if the functionality is provided by different bundles to decrease its complexity and to focus on special tasks. The intention to publish the software under an open source license is to support code contributions and to open the project for individual solutions. Moreover, the separation into bundles makes it easier for others to contribute new functionality. Further improvements will be done to optimize the current algorithms and to develop new and better filters, peak detectors and integrators.

## Conclusions

OpenChrom has been designed to become an extensible cross-platform open source software for the mass spectrometric analysis of chromatographic data. It provides extension points to enable built-in import capabilities for binary or textual instrument vendors' data formats. In addition to its custom XML format it supports the Agilent Technologies, mzXML and NetCDF mass spectrometric data format. Further development is planned to support more data formats. The open source concept has been chosen to initiate the contributions of third parties, as it depends on the ideas and needs of the community to extend the capabilities of the presented concept. OpenChrom offers extension points that enable the implementation of different baseline detectors as well as peak detectors and integrators. Furthermore, there is an option to implement filters, used to increase the chromatographic quality. The framework offers a full support of do/undo and redo operations. The examples Bioclipse and BioSunMS show how to use the Eclipse Rich Client Platform in a specific way, but no software has been published until now that is capable to import binary chromatographic files natively, offers support to edit and analyze chromatograms and makes it possible to implement new algorithms and methods. As it is open source, everybody has the possibility to inspect the implemented algorithms and methods, especially for verification. OpenChrom is a software with a special focus on the editing and evaluation of mass spectrometric chromatographic data. OpenChrom will be hopefully extended by contributing developers, scientists and companies in the future.

## Availability and requirements

**Project name: **OpenChrom

**Project homepage: **http://www.openchrom.net

**Operating systems: **Platform independent

**Programming language: **Java

**Java Runtime Environment: **Sun/Oracle JVM 1.6.0, OpenJDK

**Minimum RAM: **500 MB

**Minimum Processor: **1 GHz

**Commercial restrictions: **none

OpenChrom is available for download free of charge from the project home page.

The Agilent data file input converter must be installed separately using the OpenChrom update mechanism. The instructions how to install the converter can be found at the following website: http://www.openchrom.net/plugins/converter/agilent.

OpenChrom is licensed under the Eclipse Public License 1.0 (EPL). The EPL is an OSI approved open source license that ensures, that the source code will remain open source. OpenChrom uses some third party libraries that are partly published under different open source licenses. All third party libraries are available in separated bundles, to ensure that no license conflicts occur. The third party library bundles are published under the Apache, LGPL, AGPL and EPL license, depending on the bundle. The GPL licenses are viral, it means that derivative works must be published under the GPL license too. The EPL and Eclipse Rich Client Platform enable a different licensing for the bundles, as a bundle using methods of another bundle can not be seen as a derivative work, though it only uses its interfaces.

## Authors' contributions

PW designed and implemented the core API (Application Programming Interface), the software and its extension points. PW drafted most of the manuscript. JO gave feedback and corrected the manuscript. All authors performed extensive testing of the software and approved the final manuscript.
